# Repeated Thrombolysis Using Tenecteplase in a Patient With Early Recurrent Ischaemic Stroke

**DOI:** 10.7759/cureus.115952

**Published:** 2026-09-08

**Authors:** Lucy Carleton, Kashif Musarrat, Dominic P Dalip

**Affiliations:** 1 General Practice, Kettering General Hospital NHS Trust, Kettering, GBR; 2 Stroke Medicine, University Hospitals of Leicester NHS Trust, Leicester, GBR; 3 Internal Medicine, University Hospitals of Leicester NHS Trust, Leicester, GBR

**Keywords:** acute ischaemic stroke, early recurrent ischemic stroke, intravenous thrombolysis, repeated thrombolysis, tenecteplase (tnk)

## Abstract

Ischaemic stroke is responsible for significant morbidity and mortality. Reperfusion therapy with intravenous thrombolysis is used to improve outcomes in patients with acute ischaemic stroke within 4.5 hours of symptom onset, but is generally contraindicated in early recurrent ischaemic stroke. Despite this, cases have been reported of patients who have had repeated thrombolysis using alteplase and achieved a good outcome. Tenecteplase, a variant of alteplase with higher specificity for fibrin and a longer half-life, is increasingly used as an alternative thrombolytic agent. However, there are no previously reported cases of repeated thrombolysis using tenecteplase.

Here we report the first case of a 69-year-old male patient who received repeated thrombolysis with tenecteplase, rather than alteplase, in early recurrent ischaemic stroke.

## Introduction

Stroke is a significant cause of morbidity and mortality globally, responsible for approximately 7 million deaths and 160 million disability-adjusted life-years lost (DALYs) per year [1]. Ischaemic strokes account for around 65% of incident strokes, with around half of these occurring in people over 70 [1]. Other risk factors include smoking, excess body weight, poor diet and other metabolic risk factors such as kidney dysfunction [1].

Reperfusion therapies using intravenous thrombolytic agents, such as alteplase, are known to improve outcomes in patients with acute ischaemic stroke [2]. However, intravenous thrombolysis is generally considered to be contraindicated in patients who have had a previous ischaemic stroke within the last three months [3,4]. Despite this, there are numerous case reports of successful outcomes with repeated thrombolysis using alteplase [5-9].

Tenecteplase, a bioengineered variant of alteplase, has several advantages such as a longer half-life, greater fibrin specificity and easier administration [10,11]. It has been demonstrated to be non-inferior to alteplase [10,11], and in some cases even superior [12,13], while UK national guidelines advise that either can be used for thrombolysis in acute ischaemic stroke [14]. 

Here we report a case of a patient who was thrombolysed twice with tenecteplase after early recurrence of ischaemic stroke. The aim of this case report is to explore the safety and potential benefits of repeated tenecteplase thrombolysis in a patient who deteriorated following initial thrombolysis for acute ischaemic stroke. To the best of our knowledge, this is the first case report of a patient who received repeated thrombolysis with tenecteplase.

## Case presentation

A 69-year-old male patient presented to hospital in January 2025, one hour after sudden onset of slurred speech, facial droop and right lower limb weakness. His National Institutes of Health Stroke Score (NIHSS) [15] on admission was four due to facial palsy, right lower limb weakness and dysarthria. Prior to his admission, he was generally well with no known comorbidities. His symptoms were felt to be in keeping with a stroke, so an urgent non-contrast CT scan was done which showed no evidence of bleeding or other acute intracranial event (Figure 1). He was therefore diagnosed with a probable acute ischaemic stroke and thrombolysed with tenecteplase 90 minutes after symptom onset, at a dose of 17.5 mg (0.25 mg/kg) in accordance with national guidelines [14,16].

**Figure 1:**
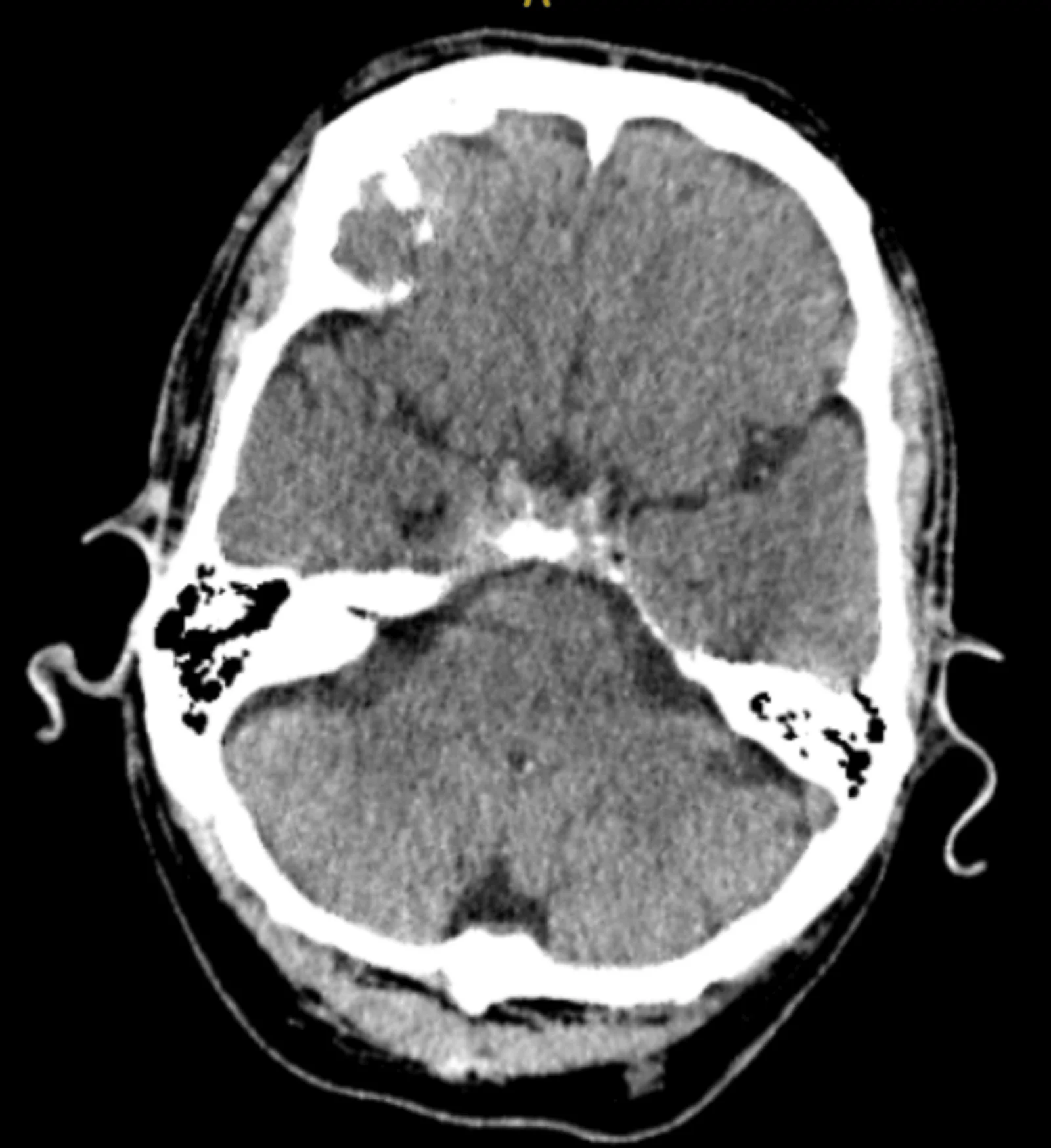
CT scan pre-thrombolysis, demonstrating no evidence of intracerebral haemorrhage

30 minutes later he developed new confusion, worsening speech problems and weakness on his right side. His NIHSS was increased to 21, as his weakness now affected his right upper and lower limbs, with worsened facial palsy, sensory deficit and severe dysarthria and aphasia. A repeat CT showed a thrombus in the M1 and proximal M2 segments of his left middle carotid artery (MCA) (Figure 2). After a thorough discussion with the patient’s family, a decision was made to thrombolyse a second time. This decision was made in part because mechanical thrombectomy was not available out of hours, while thrombolysis could be given as a rescue therapy with minimal delay. He received the same dose of tenecteplase, 2.5 hours after first thrombolysis.

**Figure 2:**
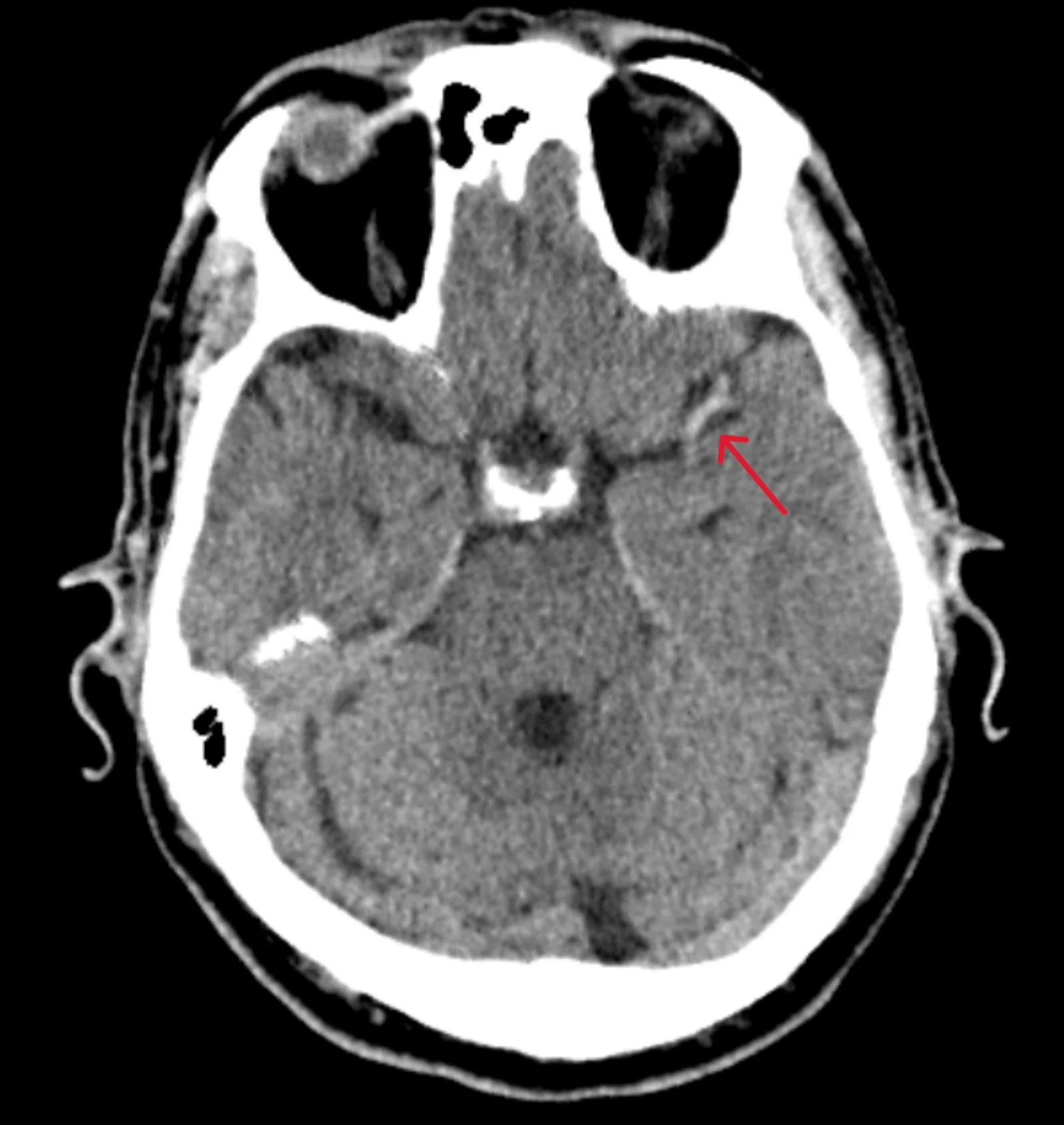
CT scan after first thrombolysis, showing a proximal MCA thrombus (red arrow) MCA: Middle carotid artery

A CT angiogram performed the next morning (13.5 hours after first symptom onset) confirmed that the thrombus in his left MCA was still present (Figures 3, 4). It also showed evidence of an acute infarct in the arterial territory with an Alberta Stroke Program Early CT Score (ASPECTS) [17] of four, so the team decided against mechanical thrombectomy.

**Figure 3:**
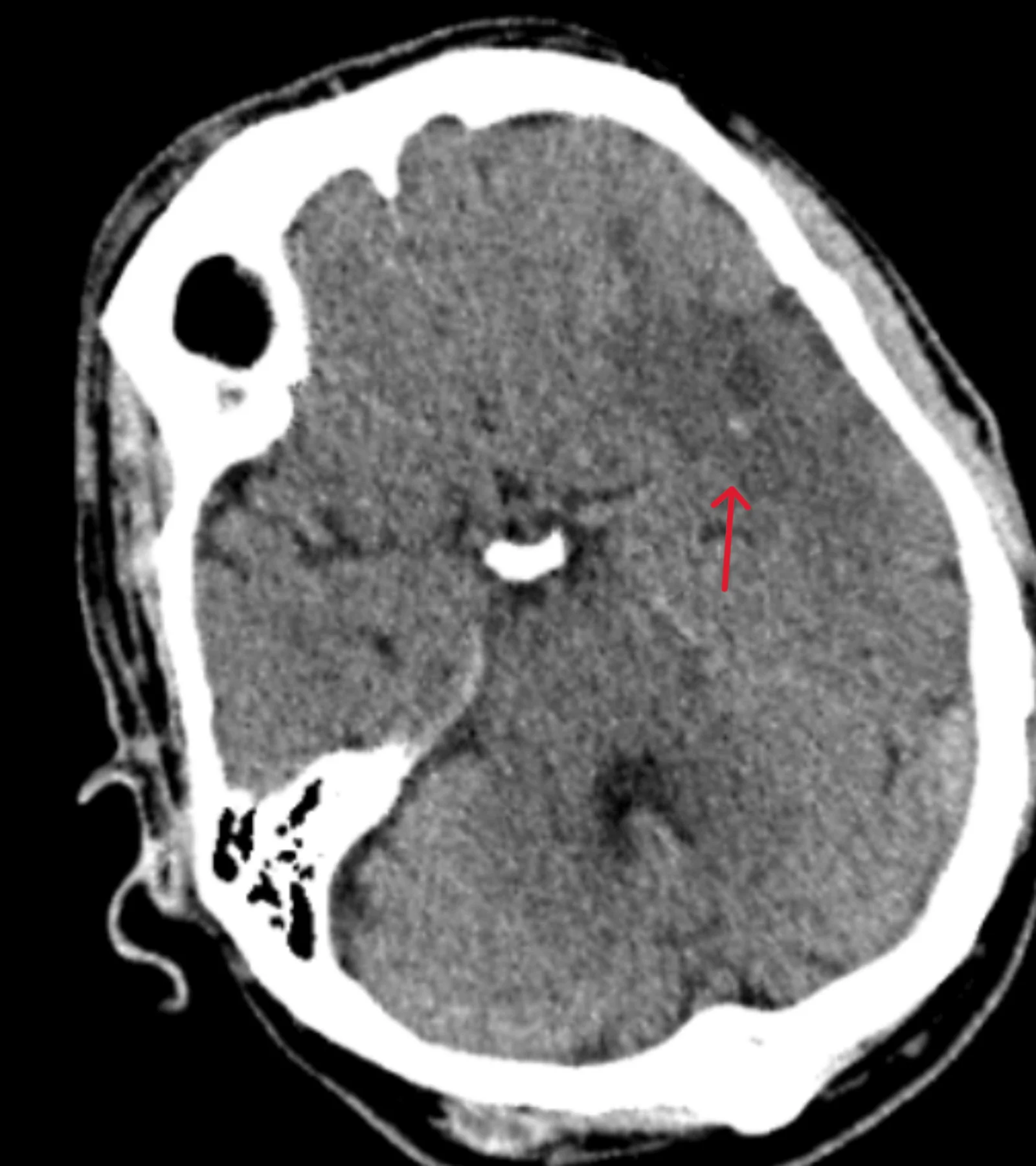
Non-enhanced CT after second thrombolysis, showing an infarcted area in the left MCA territory (red arrow) MCA: Middle carotid artery

**Figure 4:**
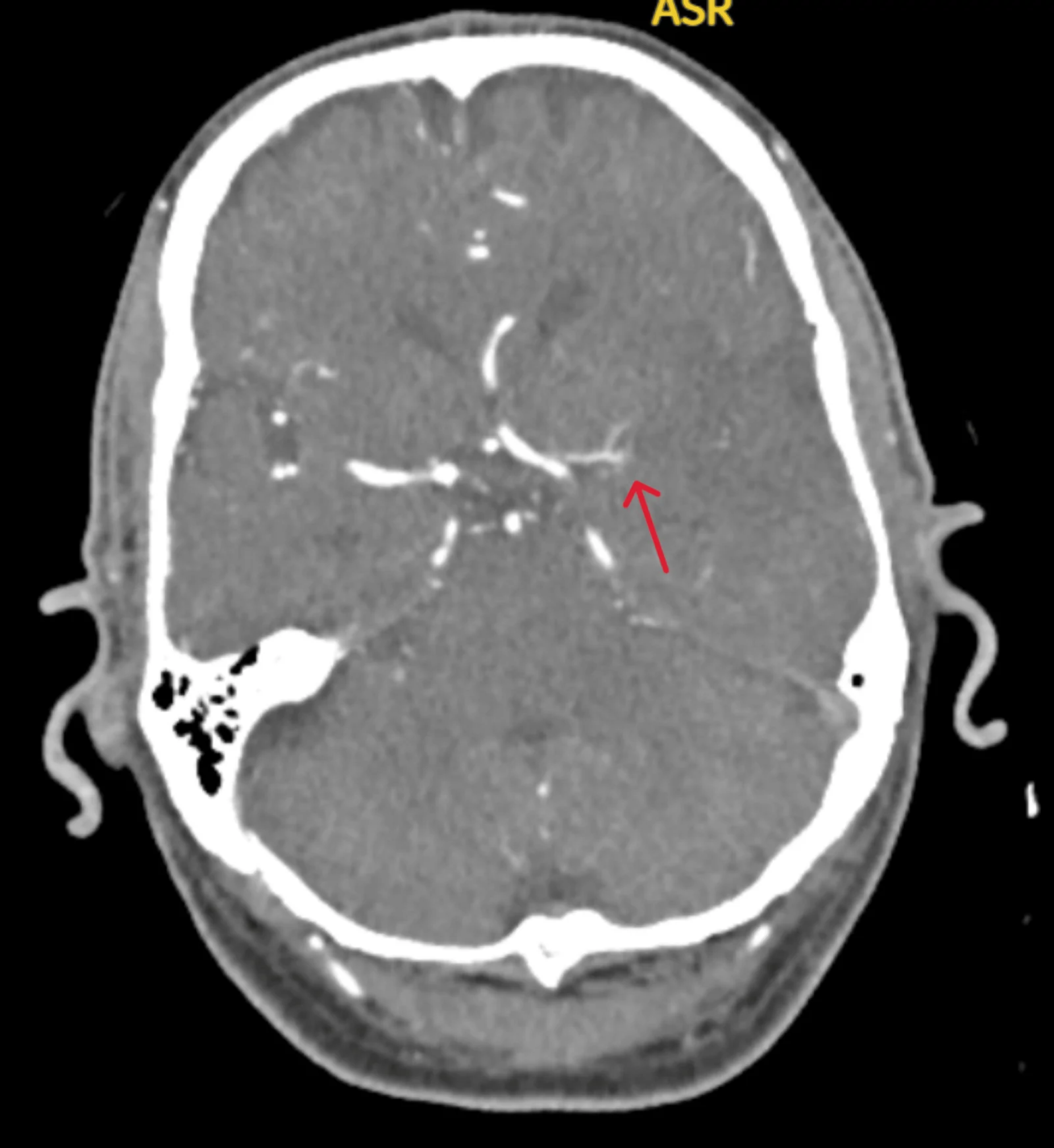
Contrast CT angiogram after second thrombolysis, demonstrating a left MCA thrombus (red arrow) with associated infarct in the arterial territory MCA: Middle carotid artery

Five days after thrombolysis, his NIHSS had improved to 11 with persistent facial and right-sided weakness but improvements to speech and sensation. During his admission he also underwent investigations for potential underlying causes of his stroke, and was found to have atrial fibrillation. He was started on edoxaban for this seven days after admission. Prior to this he had no evidence of bleeding. However, between day 5 and 15 after starting edoxaban he had three episodes of mild, self-limiting bleeding - epistaxis, haematuria and melena. 12 days after admission, he was discharged to a rehabilitation unit, where his neurological status continued to gradually improve. After his discharge from hospital, he underwent a further four months of rehabilitation in the community. At the end of this, he was able to mobilise independently due to significantly improvement in his right lower limb weakness. However, he still had minimal movement in his right upper limb, and mild to moderate expressive aphasia.

## Discussion

Intravenous thrombolysis is recommended as a treatment for acute ischaemic stroke in patients presenting within 4.5 hours of symptoms onset once intracranial haemorrhage has been excluded [2-4,14]. Multiple international guidelines recommend alteplase as the thrombolytic agent of choice [2,3], but some now acknowledge tenecteplase a non-inferior alternative [4]. The national guidelines in the UK advise that either alteplase or tenecteplase can be used, with cost being the main determinant [14]. In our local trust, tenecteplase is used over alteplase as tenecteplase requires a single dose rather than the bolus and infusion required for alteplase. This improves the ease and speed with which the thrombolytic agent can be administered.

History of ischaemic stroke within the previous three months is generally viewed as a contraindication to thrombolysis [3,4]. However, there have been multiple case reports describing repeated thrombolysis using alteplase in patients who had early recurrence of their symptoms [7,9], including in patients with underlying pathology such as a carotid web [8] or risk factors such as advanced age and low body weight [6]. A systematic review of several such case reports suggests that repeated intravenous thrombolysis with alteplase may be beneficial under certain circumstances, though with a risk of symptomatic intracranial haemorrhage [5]. However, to the best of our knowledge, there have not been any case reports of patients receiving repeated thrombolysis with tenecteplase previously.

We recount the case of a patient who was thrombolysed twice with tenecteplase after early recurrence of his ischaemic stroke. The efficacy of thrombolysis in this case was mixed. The patient’s initial symptoms resolved after the first dose of tenecteplase, but he later developed new neurological symptoms largely affecting his right side. These symptoms persisted despite the second administration of tenecteplase, and this correlated with the findings on his imaging showing that the thrombus and associated area of infarction were still present. This may have represented failed initial tenecteplase therapy, or may have been due to carotid plaque rupture, as has been reported in the literature [18].

The safety profile of tenecteplase in this case was good, as the patient suffered no intracranial or other bleeding in the first seven days after thrombolysis. After this he was started on edoxaban. However, it should be emphasised that these results are from a single case and cannot be generalised to a larger population.

There have been several reported cases of favourable functional outcomes after repeated thrombolysis with alteplase, measured by reduction in NIHSS and improvement in modified Rankin Scale [6-9,19]. However, in several cases there was either haemorrhagic transformation of the ischaemic stroke or intracranial haemorrhage, with or without symptoms [5,6,8,20]. Despite this, some studies have suggested that repeated intravenous thrombolysis is potentially beneficial in some patients with early recurrence of ischaemic stroke [5-9]. By contrast, in this case the patient experienced mixed functional outcomes, but without any bleeding sequelae until separate anticoagulation was started.

This case is notable for its use of tenecteplase rather than alteplase. Tenecteplase is a bioengineered variant of alteplase, with several advantageous features such as greater fibrin specificity, a longer half-life, and consequently easier administration (as a single dose rather than an infusion) [10,11]. Several studies have demonstrated that tenecteplase is non-inferior to alteplase in terms of functional recovery, imaging outcomes and adverse effects [10,11]. In some cases tenecteplase was found to be associated with better functional outcomes, higher odds of reperfusion and lower all-cause mortality than alteplase [12,13]. However, there is very little evidence for the use of tenecteplase in repeat thrombolysis.

## Conclusions

Evidence suggests that tenecteplase is a safe and effective alternative to alteplase for intravenous thrombolysis in ischaemic stroke, in patients without a history of recent ischaemic stroke, major bleeding or thrombolysis. While there is some evidence to support the use of alteplase for repeated thrombolysis in early recurrence of ischaemic stroke, there is still minimal evidence for tenecteplase use in these cases. This report describes a case of repeated thrombolysis with tenecteplase, but further studies are needed to explore the safety and efficacy of tenecteplase in these circumstances as this single report is insufficient to guide clinical practice.

## References

[1] Feigin VL, Brainin M, Norrving B (2025). World Stroke Organization: global stroke fact sheet 2025. Int J Stroke.

[2] Berge E, Whiteley W, Audebert H (2021). European Stroke Organisation (ESO) guidelines on intravenous thrombolysis for acute ischaemic stroke. Eur Stroke J.

[3] de Los Ríos la Rosa F, Khoury J, Kissela BM (2012). Eligibility for intravenous recombinant tissue-type plasminogen activator within a population: the effect of the European Cooperative Acute Stroke Study (ECASS) III trial. Stroke.

[4] Powers WJ, Rabinstein AA, Ackerson T (2019). Guidelines for the early management of patients with acute ischemic stroke: 2019 update to the 2018 guidelines for the early management of acute ischemic stroke: a guideline for healthcare professionals from the American Heart Association/American Stroke Association. Stroke.

[5] Wen SY, Chen FF, Chen XT, Zhang Q, Zhou CQ (2023). Repeated intravenous thrombolysis in recurrent ischemic stroke within 3 months: a systematic review. BMC Neurol.

[6] Chen F, Chen X, Zhang Q, Wen S, Zhou C (2024). Repeated intravenous thrombolysis in early recurrent ischemic stroke: a case report of an elderly low weight female. Neurol Sci.

[7] Brigo F, Bovi T, Tomelleri G, Bovi P, Moretto G (2012). Repeated systemic thrombolysis after early recurrent stroke: always hazardous?. Can J Neurol Sci.

[8] Bouchal S, Lamrani YA, Chtaou N, Maaroufi M, Belahsen F (2021). Repeated intravenous thrombolysis in early recurrent stroke secondary to carotid web: case report. Radiol Case Rep.

[9] Černík D, Ospalík D, Šaňák D, Cihlář F (2021). Repeated intravenous thrombolysis in patients with recurrent ischemic stroke in the vertebrobasilar territory. Case Rep Neurol.

[10] Rodriguez M, Sidebottom C, Wells DA, Dhasakeerthi T, Hayes L, Elangovan C, Krishnaiah B (2024). A systematic review of the efficacy and safety of tenecteplase versus alteplase in acute ischemic stroke: a time to pass the torch. Stroke Vasc Interv Neurol.

[11] Meng X, Li S, Dai H (2024). Tenecteplase vs alteplase for patients with acute ischemic stroke: the ORIGINAL randomized clinical trial. JAMA.

[12] Yogendrakumar V, Churilov L, Guha P (2023). Tenecteplase treatment and thrombus characteristics associated with early reperfusion: an EXTEND-IA TNK trials analysis. Stroke.

[13] Tsivgoulis G, Katsanos AH, Christogiannis C (2022). Intravenous thrombolysis with tenecteplase for the treatment of acute ischemic stroke. Ann Neurol.

[14] (2025). Stroke and transient ischaemic attack in over 16s: diagnosis and initial management. https://www.nice.org.uk/guidance/ng128/chapter/Recommendations.

[15] Lyden P, Brott T, Tilley B (1994). Improved reliability of the NIH Stroke Scale using video training. NINDS TPA Stroke Study Group. Stroke.

[16] (2025). Tenecteplase for treating acute ischaemic stroke. https://www.nice.org.uk/guidance/ta990/chapter/1-Recommendations.

[17] Barber PA, Demchuk AM, Zhang J, Buchan AM (2000). Validity and reliability of a quantitative computed tomography score in predicting outcome of hyperacute stroke before thrombolytic therapy. Lancet.

[18] Sallustio F, Samà D, Mascolo AP, Marrama F, Fresilli M, Diomedi M (2020). Clinical worsening despite intravenous thrombolysis in acute ischemic stroke secondary to carotid plaque rupture. J Thromb Thrombolysis.

[19] van Swieten JC, Koudstaal PJ, Visser MC, Schouten HJ, van Gijn J (1988). Interobserver agreement for the assessment of handicap in stroke patients. Stroke.

[20] Sposato LA, Salutto V, Beratti DE, Monti P, Riccio PM, Mazia C (2013). Adverse outcome of early recurrent ischemic stroke secondary to atrial fibrillation after repeated systemic thrombolysis. Case Rep Vasc Med.

